# β1 Integrin Signaling Maintains Human Epithelial Progenitor Cell Survival In Situ and Controls Proliferation, Apoptosis and Migration of Their Progeny

**DOI:** 10.1371/journal.pone.0084356

**Published:** 2013-12-27

**Authors:** Nancy Ernst, Arzu Yay, Tamás Bíró, Stephan Tiede, Martin Humphries, Ralf Paus, Jennifer E. Kloepper

**Affiliations:** 1 Department of Dermatology, University of Luebeck, Luebeck, Germany; 2 Department of Histology and Embryology, University of Erciyes, Kayseri, Turkey; 3 DE-MTA ‘‘Lendület’’ Cellular Physiology Group, Department of Physiology, University of Debrecen, Debrecen, Hungary; 4 Institute of Experimental Immunology, Euroimmun AG, Luebeck, Germany; 5 Faculty of Life Sciences, University of Manchester, Manchester, United Kingdom; 6 Institute of Inflammation and Repair, University of Manchester, Manchester, United Kingdom; National Center for Scientific Research Demokritos, Greece

## Abstract

β1 integrin regulates multiple epithelial cell functions by connecting cells with the extracellular matrix (ECM). While β1 integrin-mediated signaling in murine epithelial stem cells is well-studied, its role in human adult epithelial progenitor cells (ePCs) *in situ* remains to be defined. Using microdissected, organ-cultured human scalp hair follicles (HFs) as a clinically relevant model for studying human ePCs within their natural topobiological habitat, β1 integrin-mediated signaling in ePC biology was explored by β1 integrin siRNA silencing, specific β1 integrin-binding antibodies and pharmacological inhibition of integrin-linked kinase (ILK), a key component of the integrin-induced signaling cascade. β1 integrin knock down reduced keratin 15 (K15) expression as well as the proliferation of outer root sheath keratinocytes (ORSKs). Embedding of HF epithelium into an ECM rich in β1 integrin ligands that mimic the HF mesenchyme significantly enhanced proliferation and migration of ORSKs, while K15 and CD200 gene and protein expression were inhibited. Employing ECM-embedded β1 integrin-activating or -inhibiting antibodies allowed to identify functionally distinct human ePC subpopulations in different compartments of the HF epithelium. The β1 integrin-inhibitory antibody reduced β1 integrin expression *in situ* and selectively enhanced proliferation of bulge ePCs, while the β1 integrin-stimulating antibody decreased hair matrix keratinocyte apoptosis and enhanced transferrin receptor (CD71) immunoreactivity, a marker of transit amplifying cells, but did not affect bulge ePC proliferation. That the putative ILK inhibitor QLT0267 significantly reduced ORSK migration and proliferation and induced massive ORSK apoptosis suggests a key role for ILK in mediating the ß1 integrin effects. Taken together, these findings demonstrate that ePCs in human HFs require β1 integrin-mediated signaling for survival, adhesion, and migration, and that different human HF ePC subpopulations differ in their response to β1 integrin signaling. These insights may be exploited for cell-based regenerative medicine strategies that employ human HF-derived ePCs.

## Introduction

Integrins are transmembrane receptors that link the extracellular matrix (ECM) environment with intracellular signaling, thus regulating multiple cell functions such as cell survival, proliferation, migration, and differentiation [1–3]. 18 α and 8 β mammalian integrin subunits have been identified so far, which can assemble to 24 different heterodimers with different affinities toward specific ECM components [4–7]. The extracellular binding activity is regulated intracellularly (inside-out signaling), while extracellular binding of the ECM triggers signals that are transmitted into the cell (outside-in signaling) [6,8,9]. When specific ECM ligands bind to the extracellular region, integrin receptors cluster in the cell membrane and the cytoplasmic part of the integrin complex sends signals to the actin cytoskeleton and forms focal adhesions (FAs) [10,11]. 

Defined ECMs in adult tissues (niches) are likely to be the first molecular components interacting with stem cells (SCs) [12,13]. These niches regulate adult SC-preservation and/or differentiation and by that regulating the homeostasis of tissues/organs, like the epidermis and the cyclic hair follicle (HF) [12,14]. ß1 integrin signaling has long been thought to be important in murine epidermal and HF epithelial SCs (eSCs) [15–17]. In the HF, eSCs and partially differentiated epithelial progenitor cells (ePCs) can give rise to all epithelial cell types of the hair, the epidermis, and the sebaceous gland and are mostly found within the HF bulge [18–20]. The eSCs within this HF compartment [16,21] are slow-cycling, and show clonogenicity and proliferative capacity [22]. Potential markers for the epithelial HF SCs include β1 integrin, keratin 15 and 19 (K15, K19), α6 integrin, the transferrin receptor (CD71), p63 and CD34; however there is still considerable debate over how to distinguish the least committed, slow-cycling eSCs from their immediate progeny (i.e., rapidly proliferating, but more committed transit amplifying cells) [23–27].

Previous work has suggested that epithelial cells in human epidermis with the highest level of α2β1, α3β1 and α5β1 integrin expression show a high colony-forming efficiency (CFE) [28], and that ß1 integrin signaling is absolutely required for epidermal and HF maintenance in mice [3]. However, the role of β1 integrin signaling in human ePC maintenance or differentiation, namely in human HFs, remains to be clarified, since the bulge region of human scalp HFs does not express markedly more ß1 integrin protein than other regions of the basal layer of the human outer root sheath (ORS) [16,29].

Potential ligands for integrins expressed on HF keratinocytes are components of the basement membrane (BM) that separates the HF epithelium from its surrounding mesenchyme, the connective tissue sheath (CTS). These BM-associated integrin ligands include collagen IV, laminin-5, perlecan and nidogen [3,30]. Thus, ORS keratinocytes (ORSKs) can interact with multiple ECM components of the BM via α2β1, α3β1, and α6β4 integrins, which are differentially expressed in distinct regions of the HF [31,32]. α3β1 integrins connect the actin cytoskeleton to the BM via binding laminin-5, whereas the α2β1 integrin is found in basal keratinocytes, where it is thought to mediate cell–cell interactions and BM attachment via collagens [4,15,32].

On this background, we have further explored the role of β1 integrin-mediated signaling in the human HF epithelium *in situ*, exploiting organ-cultured human scalp HFs as an easily accessible mini-organ that represent a prototypic neuroectodermal-mesodermal interaction system in which various ePC populations can be studied within their natural tissue habitat [29,33,34]. Specifically, we wished to elucidate the impact of manipulating the outside-in signaling of β1 integrin via different ligands on the maintenance, differentiation and/or migration of distinct human ePC subpopulations *in situ*. 

This was investigated by ß1 integrin silencing in full-length human scalp HFs, which permits one to evaluate the role of ß1 integrin in ePCs within their intact human HF SC niche. In addition, dispase-pretreated organ-cultured adult human scalp HF epithelium, which lacks normal HF BM and mesenchyme, was embedded into Matrigel^®^, since the latter is rich in ECM components that are also found in the HF’s CTS and BM, such as laminin, collagen IV, heparin sulfate proteoglycans, entactin, and growth factors [12,35], with collagen I added for structural support and for mimicking any signaling input of dermal collagen. This was done in the absence or presence of activating or inhibiting ß1 integrin antibodies (see Text **S1**). Downstream ß1 integrin-mediated signaling was pharmacologically disrupted with QLT0267 (DERMIRA, [36–38]), which was developed as a putative ILK (integrin-linked kinase) inhibitor, a key adaptor protein that interacts with the cytoplasmic domains of β1 and β3 integrins and regulates many cellular processes by connecting ß1 integrin with other regulatory and adaptor proteins like Pinch, α- and β-parvins [39–41].

## Materials and Methods

### Hair follicle collection

All experiments were performed adhering to the Declaration of Helsinki Principles, and with the University of Luebeck ethics committee approval (06-109). HFs were isolated from human scalp skin obtained from routine face-lift surgery (from 12 female patients aged 19-75 years, mean age 50.2 years) after written consent. 

### β1 integrin silencing

In 3 experiments, whole HFs were cut out of human scalp skin and transfected once with a pool of 3 human *β1 integrin*-specific siRNAs (Santa Cruz, sc-35674). HFs treated with control siRNA conjugated with FITC (Santa Cruz, sc-36869) served as a scrambled control in comparison to HFs which were just cultured in transfection medium. After transfection HFs were maintained in a 6-well-plate with 3ml William’s E medium (Biochrom) supplemented with 1% L-glutamine (Invitrogen), 0.02% hydrocortisone (Sigma-Aldrich), 0.1% insulin (Sigma-Aldrich) and 1% penicillin/streptomycin mixture (Gibco, [34]). All reagents essential for transfection were obtained from Santa Cruz Biotechnology (siRNA transfection reagent, sc-29528; siRNA transfection medium, sc-36868). 

### Isolation and culturing of human hair follicles in an extracellular matrix assay

HF epithelium was isolated after dispase-pretreatment (0.1% (w/v) in William’s E medium without penicillin/streptomycin over night at 4°C and embedded into an artificial and BM mimicking ECM system, a mixture of Matrigel^®^ (BD Biosciences) and collagen I (ratio 1:1) in keratinocyte serum-free medium (K-SFM). The embedded HFs were cultured over 4 days in the presence or absence of the two different β1 integrin antibodies (an activating 12G10 and an inhibiting mAb13 antibody) or in the presence or absence of the ILK inhibitor QLT0267 in comparison to standard organ-cultured HFs (in supplemented William’s E medium) [42].

The concentration of 10µg/ml for 12G10 and mAb13 was already described as effective and established for HF organ cultures in a previous study of our lab [29] while the application of the ILK inhibitor QLT0267 concentration was chosen empirically. Adapting previous studies [36–38,43,44] which only describe different concentrations used for QLT0267 in cell culture experiments, we chose a high concentration of 100µM (dissolved in DMSO [Dimethyl sulfoxide]) being most effective in this complexity of an embedded mini-organ as the HF with its outgrowing cells. We expected that the inhibitor penetration into the tissue would be more difficult than into cultured cells. The scarcity of human HFs available for experimentation generally prohibits running of dose response studies.

During the culture we analyzed HF cell outgrowth by measuring the whole area around the HF and the two largest outgrowth points starting from the hair shaft (HF bulb, upper HF) every second day. 

### Immunofluorescence and immunohistochemical analysis

Cultured whole HFs were cryosectioned (6µm) and embedded HFs were cryosectioned (8µm). The immunoreactivity (IR) pattern of endogenous β1 integrin (12G10, 1:500; kindly given by the Humphries’ lab), keratin 15 (K15) (1:400, Chemicon, CBL), K6 (1:400, Progen), CD200-biotin (1:25, serotec), ILK (1:100, Epitomics), CD71-PE (1:100, BD Pharmingen), cleaved caspase 3 (1:400, Cell Signaling), cortactin-alexa fluor 488 (1:400, Millipore), Ki-67 and TUNEL were quantified [34]. For the quantification of the IR pattern of β1 integrin inhibitory and stimulatory antibody treated HFs a labelling of the primary antibodies, which was not already coupled with a fluorochrome or signaling molecule (biotin), was necessary. The primary antibodies against K15 and K6 were covalently attached with biotin by using the APEX^®^ Antibody Labeling Kit (Invitrogen, Biotin-XX, cat.no. A10495). 

### 5-ethynyl-2'-deoxyuridine incorporation

A more specific method to detect cell proliferation is the quantifying of only S-phase active and DNA-synthesizing cells [45]. By using 5-ethynyl-2'-deoxyuridine (EdU), a terminal nucleoside analog of thymidine, its incorporation during active DNA synthesis [46] could be visualized because of its labeling with a stabile fluorescence dye. The whole method was performed following the manufacture’s guidelines (Click-iT® EdU Alexa Fluor® 488 Flow Cytometry Assay Kit, Invitrogen).

### Quantitative real-time PCR for expression analysis of *β1* integrin*, K15,* CD200, and K6

The total RNA of 12 HFs per condition, cultured in William’s E medium or embedded in the aECM (artificial extracellular matrix) assay was extracted by using TRIreagent (Applied Biosystems/Life Technologies) and digested with recombinant RNase-free DNase-1 (Applied Biosystems) according to the manufacturer’s protocol. 1 μg of total, isolated RNA was reverse transcribed into cDNA with High Capacity cDNA kit (Applied Biosystems) following the manufacturer’s protocol. By using specific TaqMan primers and probes (Applied Biosystems, assay IDs: Hs00559595_m1 for human β1 integrin, Hs00267035_m1 for human Keratin 15, Hs01033303_m1 for human CD200 and Hs01699178_g1 for human Keratin 6) PCR amplification was performed. As internal housekeeping gene controls, transcripts of glyceraldehyde 3-phosphate dehydrogenase (GAPDH), peptidylprolyl isomerase A (PPIA) or β-actin were determined (assay IDs: Hs99999903 for human ACTB, Hs99999904 for human PPIA and Hs99999905_m1 for human GAPDH). The amount of the above mentioned transcripts was normalized to those of the control genes using the ΔCT method.

### Protein extraction and Western blot analysis of integrin-linked kinase

20 HFs were isolated after an over night 0.1% (w/v) dispase treatment (4°C) and were washed in phosphate buffered saline (PBS), flash frozen in liquid nitrogen and lysed in buffer containing 10 mM Tris-HCl, pH 7,2, 2% sodium dodecyl sulphate (SDS), 1% Triton-X100, 10% Glycerol and 2% Protease Inhibitor Set I. Later the samples were further homogenized by using ultra sound (power 60) for 15 seconds. After a centrifugation the supernatant was taken and protein concentrations were determined using the Pierce BCA protein assay kit.

20 µg protein lysates were then separated by electrophoresis through a 10% SDS gel and transferred to a PVDF (Polyvinylidene Difluoride) membrane. The membrane was blocked in 5% non-fat dried milk in tris buffered saline (TBS), 0.05% Tween-20, incubated in primary antibody (ILK 1:2000, Epitomics, β-actin 1:1000, Santa Cruz) overnight at 4°C, washed in TBS/0.05% Tween-20, and incubated with horseradish peroxidase (HRP)-conjugated secondary antibody (Jackson Immunoresearch Laboratories) for 1h at room temperature. Secondary antibody was visualized using ECL (enhanced chemiluminescence) reagent, according to the manufacturer's instructions (GE Healthcare). 

### Statistical analyses

All data were given as the mean of normalized data±SEM (standard error of the mean) and the evaluation of statistical significance was performed by using GraphPad Prism 5.01 (Graph Pad software, Inc., San Diego, CA, USA). T-test or one-way ANOVA followed by an appropriate post hoc comparison (depending on a given Gaussian distribution) was used specifically.

## Results

### β1 integrin silencing reduces proliferation and DNA synthesis in different progenitor cell populations of the human hair follicle epithelium

**Figure 1:**
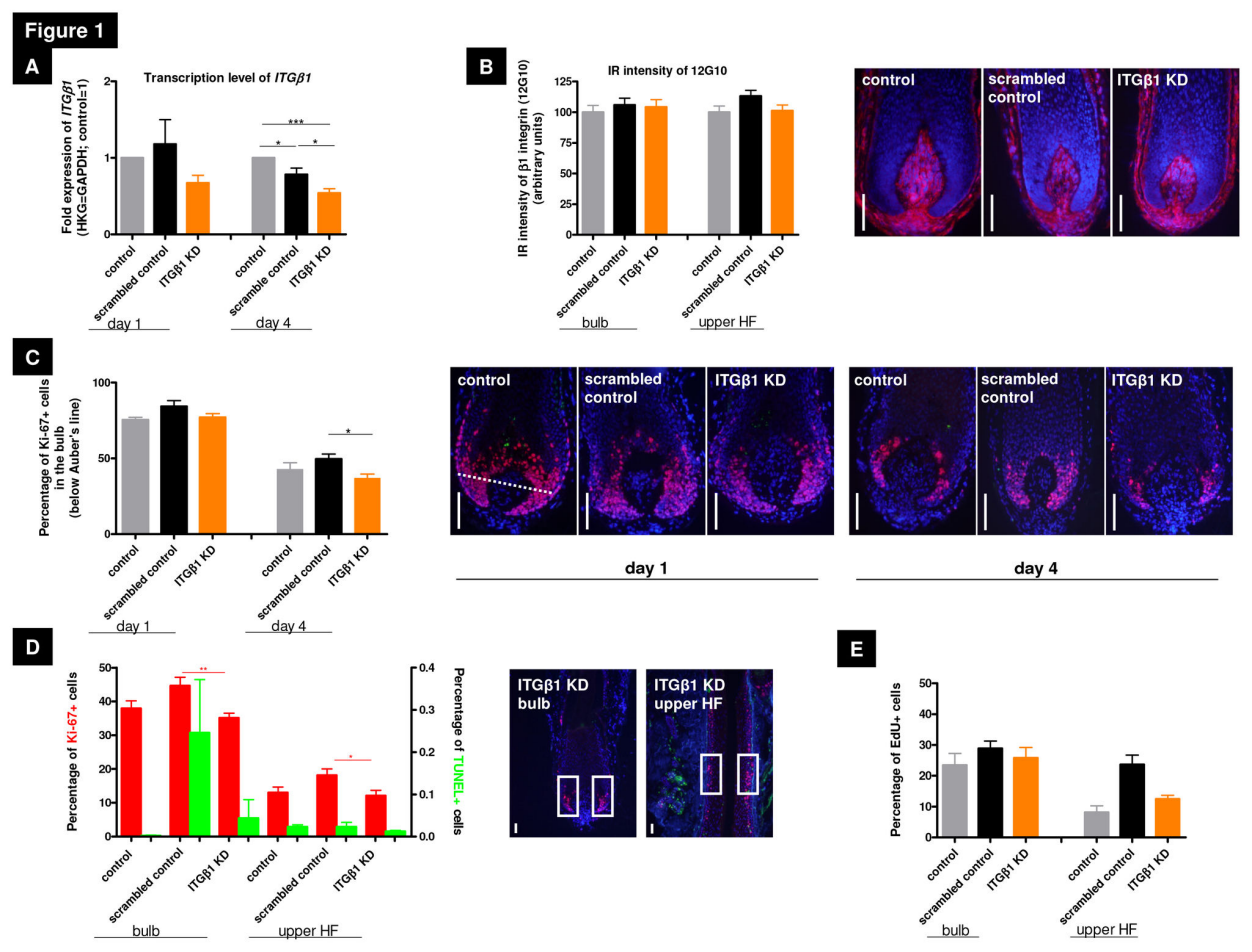
Gene silencing of *β1* integrin in normal human hair follicles. (A) *ß1* integrin gene expression was analysed with qRT-PCR using full-length hair follicles (HFs). At day1 the silencing had a strong influence on the *ß1* integrin transcription of HFs, PCR results on day 4 confirmed a significant silencing. Fold expression of all analyzed genes were normalized to GAPDH. n =2-3 individuals (for RNA extraction 12 HFs/patient were used and cultured over 4 days). (B) The immunoreactivity (IR) pattern of ß1 integrin in the whole HF was analyzed on day4 using the ß1 integrin-activating antibody 12G10. The IR intensity displayed no differences in the analyzed groups and in the different HF compartments. The control is normalized to 100%. n=17-26 HFs of 3 individuals; representative photos of HF bulbs on day4. (C) *β1* integrin silencing caused a significant reduction of Ki-67^+^ matrix keratinocytes of anagen HFs (counted below Auber’s line; dotted white line) treated with *β1* integrin siRNA compared to the scrambled control on day4. n=13-16 HFs of 3 individuals. (D) To dissect the proliferation capacity of slow-cycling epithelial progenitor cells of the HF bulge Ki-67^+^ cells were counted in rectangles (representative photos). *β1* integrin silencing caused a significant reduction of Ki-67^+^ cells in the bulb, but also in the HF bulge on day4. n=13-24 HFs of 3 individuals. Red bars=Ki-67, green bars=TUNEL. (E) By analyzing the proliferating status in a human HF via counting EdU^+^ cells in our rectangles we could show the same tendency for proliferation in β1 integrin-mediated signaling as counting Ki-67^+^ cells in the analyzed HF compartments. n=2-3 HFs of 1 individual. White scale bars represent=50µm. All statistical analyses were performed with the one-way ANOVA by appropriate post hoc comparison (depending on a given Gaussian distribution), mean of normalized data +/- SEM (*p<0.05, **p<0.01, ***p<0.001). Abbreviation: HKG=housekeeping gene, GAPDH=glyceraldehyde3-phosphate dehydrogenase, ITGβ1 KD=knock down of ß1 integrin, IR=immunoreactivity.

β1 integrin is prominently expressed throughout the human HF BM, including the eSC niche region of the HF [16,17], and human HF ePCs from the bulge overexpress *β1 integrin* mRNA [47,48]. Therefore, we first investigated the overall effects of *β1 integrin* silencing on human ePC functions *in situ* within intact, full-length, organ-cultured human scalp HFs that had been transfected with a cocktail of three *β1 integrin*-specific siRNAs or with scrambled control RNAs, using a standardized method that had previously been found to be effective [49,50] following the manufacturer’s guidelines (Santa Cruz). Successful *β1 integrin* knock down in human anagen scalp HFs was demonstrated at the mRNA level on day 4 (Figure **1A**), yet did not change ß1 integrin protein IR in the silenced HFs compared to controls after 4 days (Figure **1B**).

The mammalian HF epithelium harbors different progenitor cell populations with distinct proliferation capacities, such as slow-cycling, intermittently proliferating ePC populations in the bulge versus rapidly proliferating, transit amplifying cells in the hair matrix [51,52]. Analyzing only anagen VI HFs, quantitative immunohistomorphometry of the proliferation marker Ki-67 showed that, compared to scrambled control HFs, *β1 integrin*-specific silencing significantly reduced the number of Ki-67^+^ cells (10% less than scrambled control) in the maximally proliferating hair matrix (Figure **1C**), and also significantly reduced the number of slow-cycling Ki-67^+^ cells in the HF bulge (Figure **1D**). These results were double-checked by measuring EdU incorporation, a cell cycle S-phase specific marker to determine active DNA synthesis [46]. Counting EdU^+^ cells in defined reference areas in the HF bulb and HF bulge, the same proliferation-inhibitory tendency after *β1 integrin* silencing could be demonstrated in both HF compartments (Figure **1E**, Figure **S2C**). Instead, *β1 integrin* knock down did not significantly affect apoptosis in the HF bulb, as measured by TUNEL assay (Figure **1D**). 

Thus, even though *β1 integrin* knock down was documented only on the mRNA level (perhaps due to extended ß1 integrin protein stability within the human HF), silencing was functionally effective since it reduced proliferation and DNA synthesis in both slow-cyling human bulge ePCs and rapidly proliferating human hair matrix keratinocytes *in situ*. This suggests that β1 integrin may indeed operate as an important niche receptor that regulates proliferation activity in different ePC populations in the human HF. 

### β1 integrin-mediated signaling is required for human epithelial progenitor cell maintenance *in situ*

**Figure 2:**
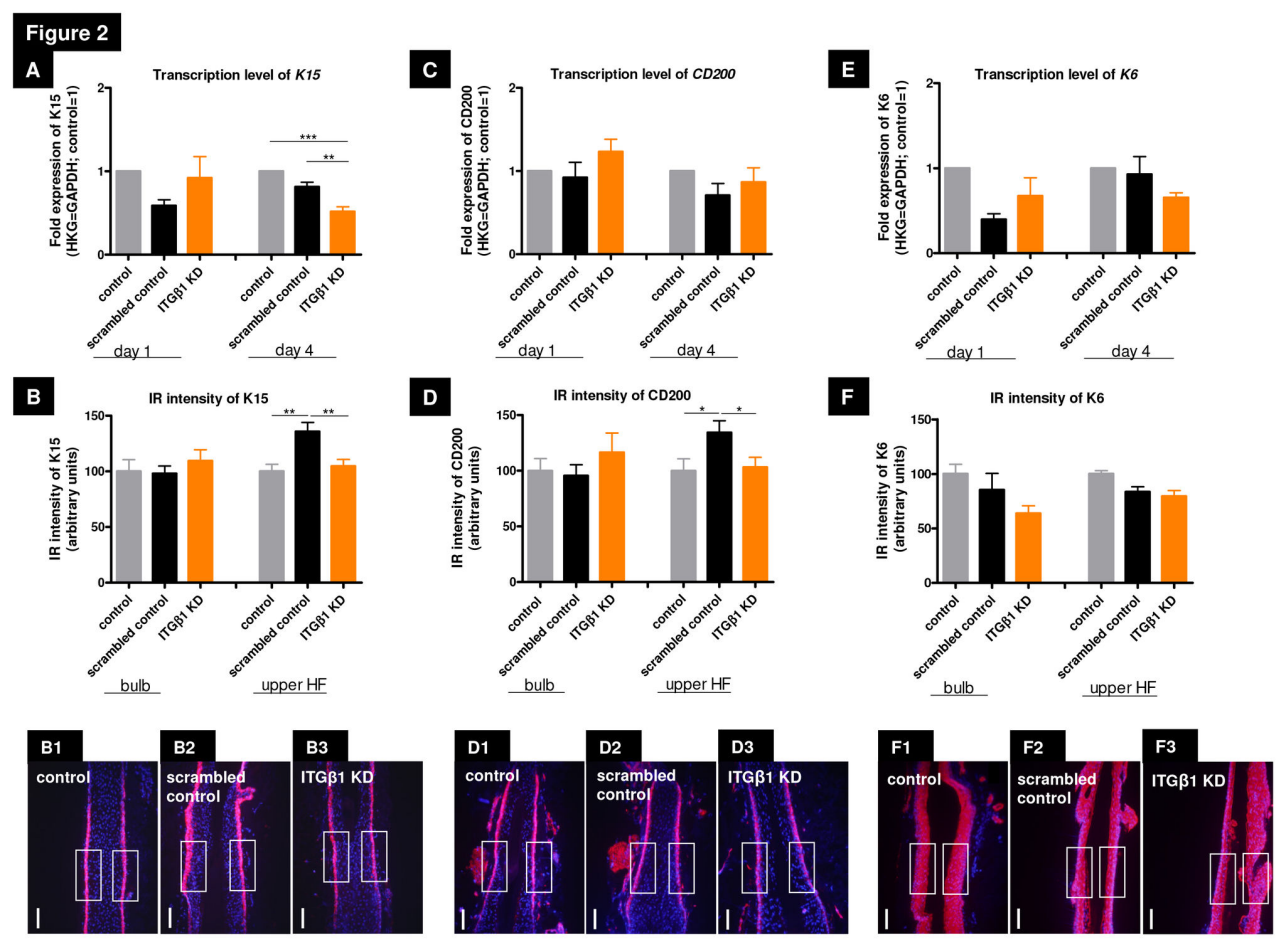
β1 integrin silencing reduced significantly the epithelial progenitor protein expression in the hair follicle bulge. (A) Keratin 15 (K15) transcription was significantly reduced at day 4 by *β1* integrin siRNA compared to the scrambled control. (B) K15 immunoreactivity (IR) was most downregulated in *β1* integrin siRNA silenced HFs in the upper HF (including the bulge region), n=19-26 HFs of 3 individuals. (B1-3) Representative photos demonstrating the reference areas in the upper HF. (C) *β1* integrin silencing slightly enhanced the gene expression of CD200. (D) CD200 IR in the HF bulge was significantly reduced compared to the scrambled control at day 4; n=17-28 HFs of 3 individuals. (D1-3) Representative photos which show the reference areas in the upper HF. (E) *β1* integrin knock down initially enhanced expression of K6, but reduced the transcription level subsequently at day 4. (F) IR intensity of K6 in the different HF compartments. It demonstrated a non-specific repression of K6 IR in every analyzed HF compartment by the silencing procedure as such, but not specifically by *β1* integrin silencing. n=16-18 HFs of 2 individuals. (F1-3) Representative photos which show the reference areas in the upper HF. Fold expression of all analyzed genes were normalized to GAPDH. n=2-3 individuals (for RNA extraction 12 HFs/patient were used and cultured over 4 days). IR intensity of the HF bulb and the upper HF was measured with a specified rectangle with ImageJ (250x125). White scale bars represent=100µm. All statistical analyses were performed with the one-way ANOVA by appropriate post hoc comparison (depending on a given Gaussian distribution), mean of normalized data +/- SEM (*p<0.05, **p<0.01, ***p<0.001). Abbreviation: HKG=housekeeping gene, GAPDH=glyceraldehyde 3-phosphate dehydrogenase, ITGβ1 KD=knock down of *ß1* integrin, K=keratin, IR=immunoreactivity.

To determine if β1 integrin-mediated signaling is needed for the maintenance and differentiation of ePCs, we analysed the effects of *β1 integrin* knock down on the expression of ePC markers K15 and CD200 [16,53,54] in human HFs *in situ*. Initially, i.e. one day after knock down, *β1 integrin* silencing even slightly enhanced *K15*, *CD200* and *K6* gene expression in human scalp HFs (Figure **2A,C,E**), possibly as a temporary compensatory phenomenon. Subsequently, however, K15 transcription was significantly reduced 4 days after silencing by *β1 integrin* siRNA compared to scrambled controls (Figure **2A**). In the main eSC region, the HF bulge, this was also associated with a significant reduction of K15 and CD200 protein IR (Figures **2B,B1**-**3**, Figures **2D,D1**-**3**), suggesting that uninterrupted ß1 integrin signaling is required to maintain human HF eSCs within their niche.

We then investigated if *β1 integrin* silencing impacts on the expression of K6, which is prominently and constitutively expressed by differentiated keratinocytes throughout the human ORS, but not by HF bulge eSCs [55,56] and on CD71 expression, a marker of transit amplifying cells, the immediate progeny of eSCs [57,58]. After *β1 integrin* silencing a non-specific repression of K6 IR in both HF compartments (Figure **2E,F**) can be seen and CD71 protein IR was only slightly reduced (Figure **S2A**). While ß1 integrin-mediated signaling is required to preserve the adult ePC pool in adult human HFs, a major overall differentiation-modulatory impact of *β1 integrin* silencing could not be confirmed for K6 and CD71*.*

### ß1 integrin silencing does not alter expression of the hair follicle bulge immune privilege marker MHC class Ia

The prominent expression of the immunoinhibitory “no danger-signal”, CD200 in the HF bulge [59] not only demarcates ePCs [16,21,53], but also constitutes part of the relative immune privilege of the HF bulge, which may protect the HF eSC niche against autoimmune attacks and is characterized by an extremely low expression of major histocompatibility complex (MHC) class Ia [21,60,61]. While CD200 IR in the HF bulge was significantly reduced (Figures **2D,D1**-**3**), *β1 integrin* silencing did not alter the (already minimal) MHC Ia IR within the human HF bulge (Figure **S2B**). This suggests that intact ß1 integrin signaling is not essential for the maintenance of the MHC class Ia-based immune privilege of the human bulge.

### β1 integrin ligands enhance human hair follicle keratinocyte outgrowth *in situ*

**Figure 3:**
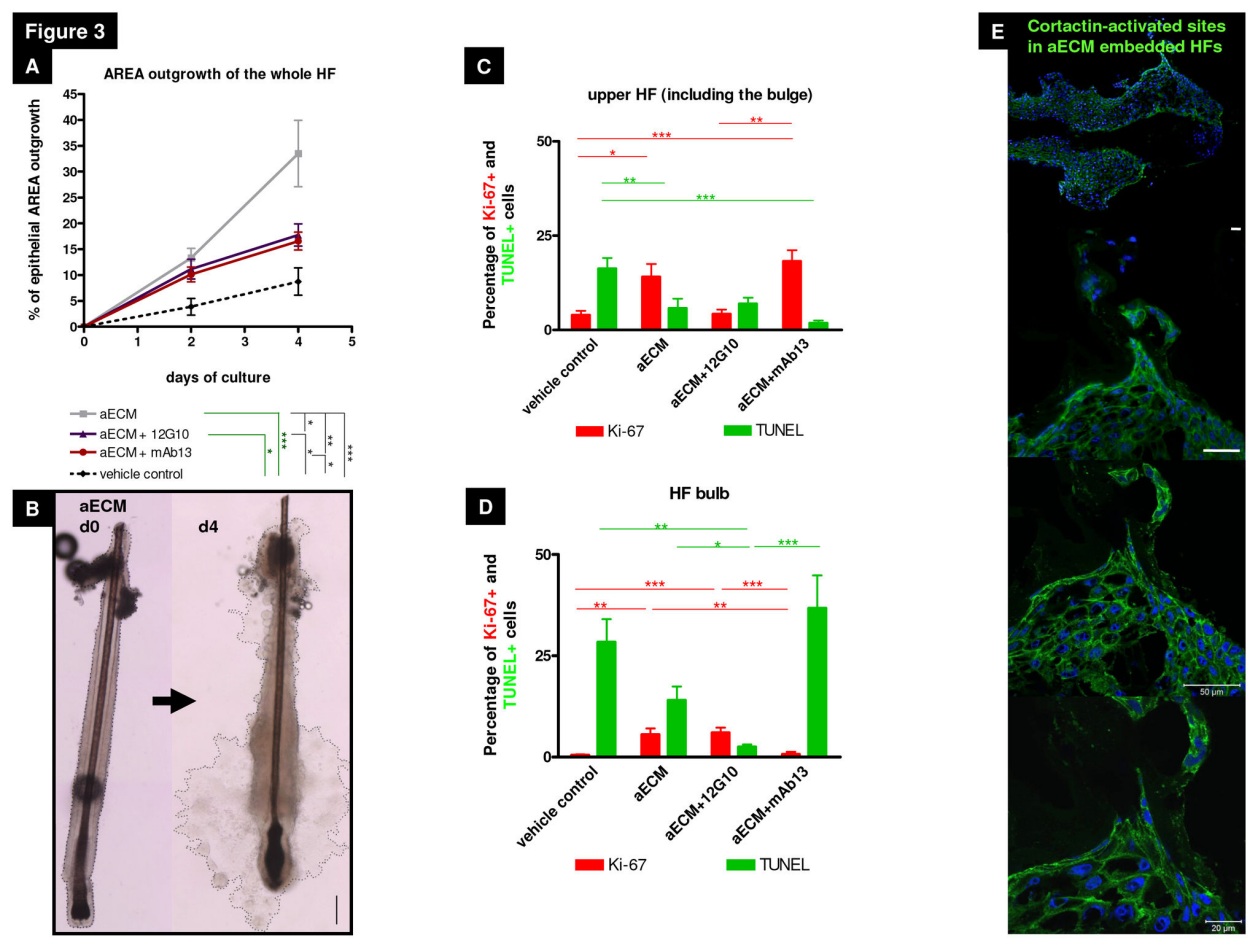
β1 integrin receptor ligands differentially regulate vitality of keratinocytes in different hair follicle compartments. (A) Epithelial outgrowth area of outer root sheath keratinocytes (ORSKs) over 4 days was measured. While the vehicle control hair follicles (HFs) showed no ORSK outgrowth in the culture dishes, the embedded HFs (aECM) showed a 30% larger ORSK outgrowth area. Activating and inhibiting β1 integrin antibodies had very similar stimulatory effects on ORSK outgrowth area. n=20-41 HFs of 3-4 individuals. Green lines and stars mark the significances of day2; black lines and stars mark the significances of day4. Statistical analysis was performed with the Mann-Whitney test; mean+/-SEM (*p<0.05, **p<0.01, ***p<0.001). (B) Representative photos of dispase-treated, embedded HF (aECM). Dotted line demarcates the area of analysis for the ORSK outgrowth over the culture period of 4 days. Black scale bar=100µm. (C) Ki-67/TUNEL-staining demonstrated that β1 integrin ligands, like extracellular matrix components and the specific receptor antibodies, decreased apoptosis in the upper HF, whereas in the aECM and aECM+mAb13-treated group the proliferation rate is up-regulated, aECM+12G10 is similar to the vehicle control in the HF bulb. n=7-15 HFs of 2-3 individuals. (D) Ki-67/TUNEL-staining confirmed the influence of β1 integrin ligands on HF bulb cells. In the aECM and aECM+12G10-treated group the number of proliferative cells in the human HF bulb significantly increased. The inhibiting antibody mAb13 enlarged apoptosis in HF bulb cells. n=8-16 HFs of 2-3 individuals. Statistical analyses of Ki-67/TUNEL were performed with the one-way ANOVA by appropriate post hoc comparison (depending on a given Gaussian distribution), error bars=mean of normalized data +/- SEM (*p<0.05, **p<0.01, ***p<0.001). Red bars=Ki-67, green bars=TUNEL. (E) Cortactin revealed activated migration mainly in the HF bulb of the aECM-treated group. White scale bars=50µm. Abbreviation: aECM=artificial ECM consisting of Matrigel^®^, collagen I and K-SFM (keratinocyte-serum free medium), aECM+12G10=aECM supplemented with the activating β1 integrin antibody 12G10, aECM+mAb13=aECM supplemented with the inhibiting β1 integrin antibody mAb13.

Next, we investigated the influence of ECM ligands of the human HF mesenchyme (BM, CTS) that are likely to interact with ß1 integrin. For this purpose, human scalp HFs were treated with dispase, which cleaves collagen IV and fibronectin [62,63] to remove the HF BM and CTS. The remaining denuded HF epithelium was then embedded into an ECM environment that partially mimics aspects of the native HF mesenchyme and BM (i.e. Matrigel^®^, which is rich in the ß1 integrin ligands laminin, collagen IV, heparin sulfate proteoglycans, entactin, and growth factors [12,35,64], combined for greater stability with collagen I which represents the main dermal collagen). Both components were diluted in K-SFM, which is optimized for the isolation and expansion of human keratinocytes [65]. The outgrowth of ORSKs from plated human HF epithelium was measured planimetrically during three different time points. This demonstrated that only the HFs embedded into an aECM, consisting of Matrigel^®^ and collagen I, showed marked ORSK outgrowth of the whole HF area (Figure **3A,B**). This suggests that ECM-mediated signaling via integrins expressed on ORSKs is indispensable for ORSK migration *in situ*. 

Interestingly, the addition of anti-β1 integrin antibodies (namely, the specific ß1 integrin activating [12G10] or inhibiting [mAb13] antibodies [66,67]) enhanced the ORSK outgrowth area compared to the dispase-pretreated HF epithelium not embedded into aECM (“vehicle control”), but it showed significantly less outgrowth compared to denuded HFs embedded in aECM. Surprisingly, both activating and inhibiting ß1 integrin antibodies had very similar stimulatory effects on the ORSK area outgrowth (of the whole HF) (Figure **3A**).

### β1 integrin receptor ligands differentially regulate epithelial cell proliferation and apoptosis in distinct human hair follicle compartments

Since outside-in signaling via β1 integrin regulates many fundamental epithelial cell functions [6,68], we sought to correlate the observed differences in ORSK outgrowth to proliferation and apoptosis markers. When the dispase-pretreated HF epithelium embedded in the CTS- and BM- mimicking aECM was compared with standard organ-cultured, but also dispase-pretreated HFs, removal of the BM and CTS promoted epithelial cell apoptosis in the human HF epithelium *in situ*. The contact of dispase-pretreated HFs with the aECM alone already significantly reduced apoptosis and up-regulated proliferation of the HF epithelium (Figure **3C,D**). Notably, the number of proliferating cells in the upper HF epithelium was 3 times higher than in the HF bulb. This suggests that the composition of aECM activated the outside-in signaling mediated by β1 integrin and thus prolonged survival of the embedded HF epithelium; moreover, this enhanced the proliferation rate in the HF bulge, the SC-rich and slow-cycling HF compartment.

**Figure 4:**
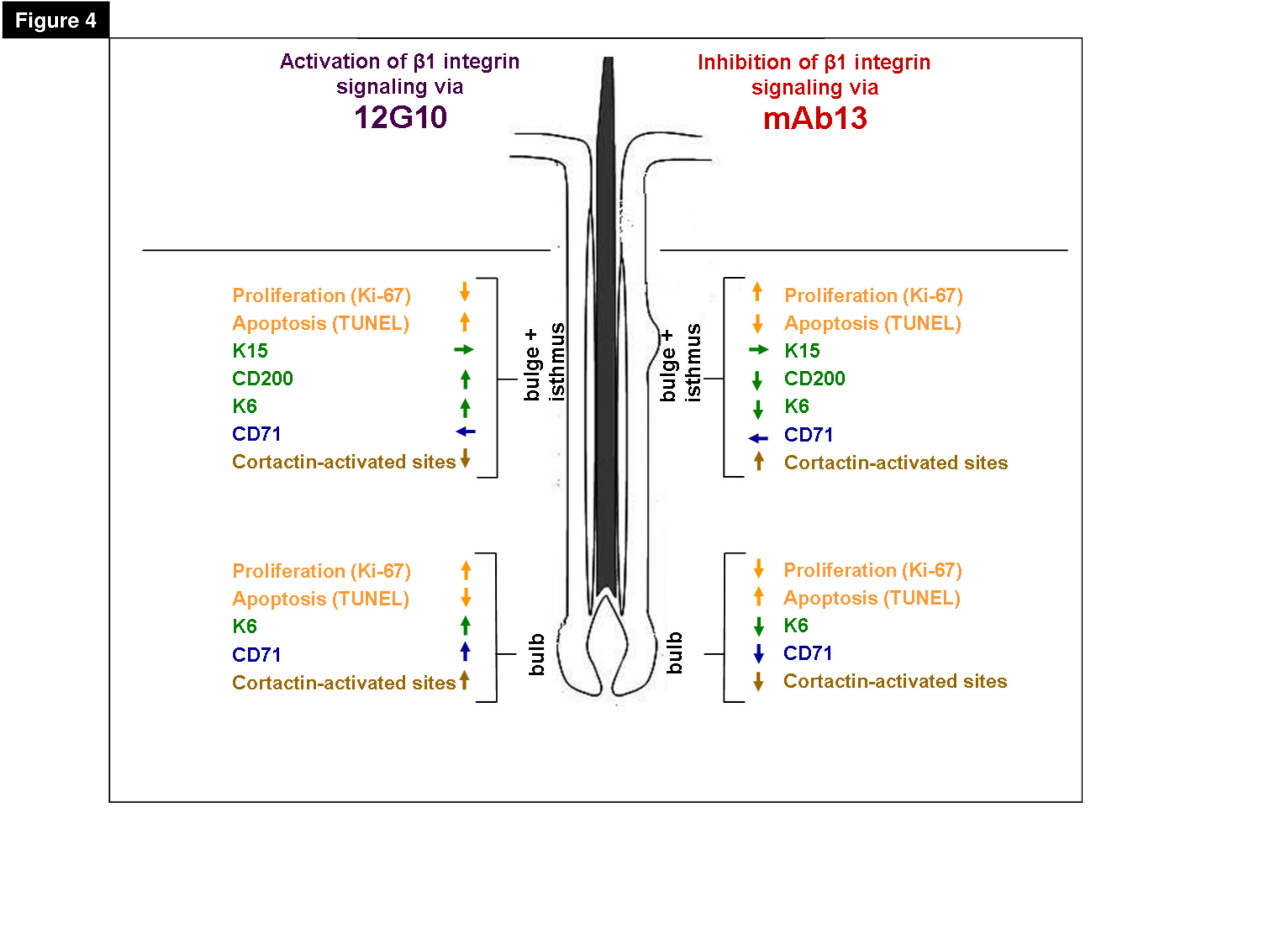
Schematic drawing for differential regulation via β1 integrin antibodies. Comparison of the influences of activated and inhibited signaling via β1 integrin specific antibodies on the protein expression of different immunoreactivity markers. The immunoreactivity analyses of the β1 integrin-activating (12G10) or –inhibiting (mAb13) antibody-treated and aECM (artificial extracellular matrix medium) embedded hair follicle (HF) epithelium suggested a different response of the epithelial progenitors cell subpopulations on ß1 integrin signaling. The application of aECM-incorporated β1 integrin antibodies allowed distinguishing adult human epithelial progenitor cell subpopulations with distinct amplifying capacities *in* *situ*, which are located in separate epithelial compartments of human scalp HFs.

Testing, next, the effects of β1 integrin antibodies incorporated into aECM showed that the ß1 integrin-stimulatory antibody (12G10) significantly reduced apoptosis in the HF bulb, and reduced proliferation in the upper HF compared to the aECM group (Figure **3C,D**). Instead, the ß1 integrin-inhibitory antibody (mAb13) had the opposite effect and up-regulated apoptosis, yet only in the hair bulb; unexpectedly, it induced proliferation in the upper HF compartments including the bulge (Figure **3C,D**, Figure **4**). These antibody stimulation experiments suggest that distinct human ePCs in the HF show a differential proliferation/apoptosis response *in situ* to ß1 integrin-mediated signaling.

### The extracellular matrix environment stimulates hair follicle keratinocyte migration primarily in the hair bulb

Besides proliferation and apoptosis, ORSK outgrowth is likely to be dominated by ORSK migration events. This was gauged by cortactin immunohistomorphometry, since activated cortactin accumulates in actin-enriched lamellipodia and membrane ruffles at the moving edge of migrating epithelial cells, signifying a role in actin network formation [69].

The hair bulbs of dispase-pretreated and subsequently aECM-embedded HFs showed strong activated cortactin IR, which was prominently expressed in a larger number of FA-like structures [70]. ORSKs showed enhanced migration into the provided aECM. This may explain why the largest outgrowth of ORSKs was measured around the HF bulb (Figure **3E**) although the highest proliferative (Ki-67^+^) capacity of ORSKs was mainly seen in the upper HF (Figure **3C**). Therefore, the massive ORSK outgrowth seen in our CTS- and BM- mimicking ECM system likely also enhanced ORSK migration in the presence of ß1 integrin ligands. 

### Different human epithelial progenitor cell populations differ in their dependence on β1 integrin signaling *in situ*

To probe, next, whether β1 integrin-mediated signaling is really needed for the maintenance and differentiation of ePCs we checked the effects of *β1 integrin* knock down on the expression of ePC markers K15 and CD200 [16,47,54,71] were analyzed on the gene and protein expression level (Figure **S1A-F**). For qRT-PCR the entire dispase-pretreated, embedded and cultured HF epithelium was used. The upper HF including the bulge showed that the HF-ECM mimicking system significantly down-regulated the expression of the ePC markers K15 and CD200 on the gene and protein level in contrast to the vehicle control (dispase-pretreated HF epithelium cultured without Matrigel^®^/collagen I) (Figure **S1A,B,D,E**). 

The dispase-pretreated HF epithelium embedded in aECM or embedded and 12G10-treated showed a reduction of the transcription level of *K6* (Figure **S1C**), while the K6 IR pattern demonstrated a strong differentiation-inducing capacity in the whole HF epithelium (Figure **S1F**). Opposite results with regard to K6 qRT-PCR and IR were obtained for the standard denuded HF epithelium (vehicle control) compared to denuded, aECM embedded and mAb13-treated HFs (Figure **S1C,F**). Thus, although our CTS- and BM- mimicking ECM components, which are expected to mimic endogenous β1 integrin ligands, optimize the survival of HF epithelium, the same ligands reduce the ePC reservoir in the human HF bulge and push this rapidly proliferating compartment of the HF epithelium towards differentiation, as indicated by increased K6 protein expression.

### Inhibiting or activating β1 integrin signaling differentially stimulates human epithelial progenitor cells and their progeny in distinct hair follicle compartments

**Figure 5:**
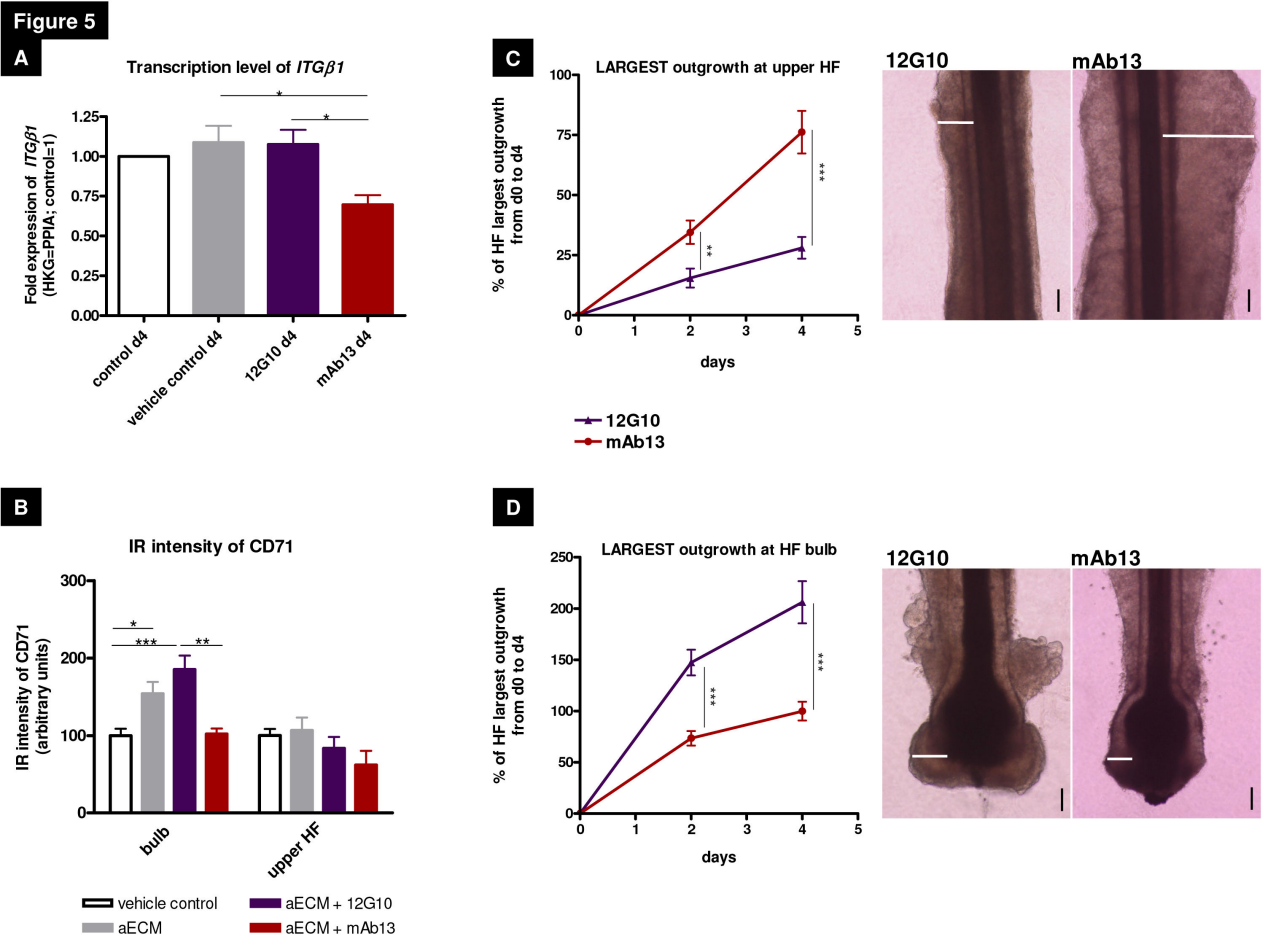
β1 integrin-mediated signaling stimulated different epithelial progenitor cell populations in distinct hair follicle compartments. (A) Relative gene expression of *β1* integrin. The β1 integrin-activating antibody 12G10 did not alter β1 integrin gene expression, whereas the inhibition of the receptor via mAb13 significantly reduced β1 integrin expression. n=1-2 individuals in experimental triplicates (15 hair follicles [HFs]). (B) Immunoreactivity of CD71 enabled to distinguish between different epithelial progenitor cell populations within the HF bulge and the HF bulb. n=5-10 HFs of 3-4 individuals. The data were analysed by using the one-way ANOVA with the appropriate post hoc comparison (depending on a given Gaussian distribution), mean of normalized data +/- SEM (*p<0.05, **p<0.01, ***p<0.001). (C) Measurement of the largest outgrowth in the upper HF and (D) HF bulb over 4 days displayed the large influence of the β1 integrin antibody treatment and distinguished between the different epithelial progenitor cell populations via their response to β1 integrin antibody stimulation. In the upper HF region (including the bulge) the inhibiting β1 integrin antibody mAb13 significantly stimulated epithelial outgrowth, whereas in the HF bulb the activating antibody 12G10 antibody stimulated epithelial outgrowth. Photos show dispase-pretreated upper HFs and HF bulbs after embedding into the aECM (artificial extracellular matrix) system and treating with β1 integrin antibodies at day 4. White lines demarcate the reference areas. n=18-33 HFs of 4 individuals. Mean+/-SEM, using unpaired t-test (*p<0.05, **p<0.01, ***p<0.001). Scale bars: 100µm. Abbreviation: aECM=artificial ECM consisting of Matrigel^®^, collagen I and K-SFM (keratinocyte-serum free medium), aECM+12G10=aECM supplemented with the activating β1 integrin antibody 12G10, aECM+mAb13=aECM supplemented with the inhibiting β1 integrin antibody mAb13, HKG=housekeeping gene, PPIA=peptidylprolyl isomerase A, IR = immunoreactivity.

Since this had never been tested before in human epithelium *in situ*, we also wanted to examine if anti-integrin antibodies impact on *β1 integrin* transcription in adult human scalp HFs *in situ*. qRT-PCR showed that the aECM-incorporated stimulatory β1 integrin antibody (12G10) demonstrated no further upregulation on day 4 in comparison to the aECM group (Figure **5A**). Instead, the inhibitory mAb13 antibody down-regulated *β1 integrin* gene expression in human HFs *in situ* (Figure **5A**). This is the first demonstration of a direct transcriptional effect of the inhibitory antibody on *β1 integrin* gene expression in a human mini-organ. 

Next, we tested whether distinct subpopulations of human ePCs and their progeny *in situ* showed a differential response pattern to the stimulation with antibodies that either stimulate or inhibit ß1 integrin-mediated signaling [29,72–74]. Indeed, this was the case. While the whole area outgrowth measurements of the HFs did not show stimulatory or inhibitory differences (Figure **3A**), the proliferation and apoptosis analyses demonstrated significant differences in the HF bulb and the upper HF (Figure **3C,D**). This was confirmed by corresponding differences in ORSK largest outgrowth in these two defined HF compartments (Figure **5C,D**). While the ß1 integrin activating antibody 12G10 enhanced ORSK largest outgrowth mainly in the HF bulb (Figure **5D**), interestingly and unexpectedly, in the upper HF (including the bulge), epithelial cell largest outgrowth was stimulated by the inhibitory antibody mAb13 (Figure **5C**). Moreover, studying expression of CD71 the activating antibody 12G10, but not the inhibitory mAb13, significantly enhanced CD71 IR (and thus the number of transit amplifying cells) in human HF bulbs (Figure **5B**; see also Figure **4** and Text **S2**). This suggests, dependent on the analysis method (area or largest outgrowth) that inhibiting or activating β1 integrin signaling elicits differential responses in adult human HF ePCs compared to their more committed epithelial progeny*.*

### QLT0267 impacts on β1 integrin-mediated signaling in human hair follicle epithelium

**Figure 6:**
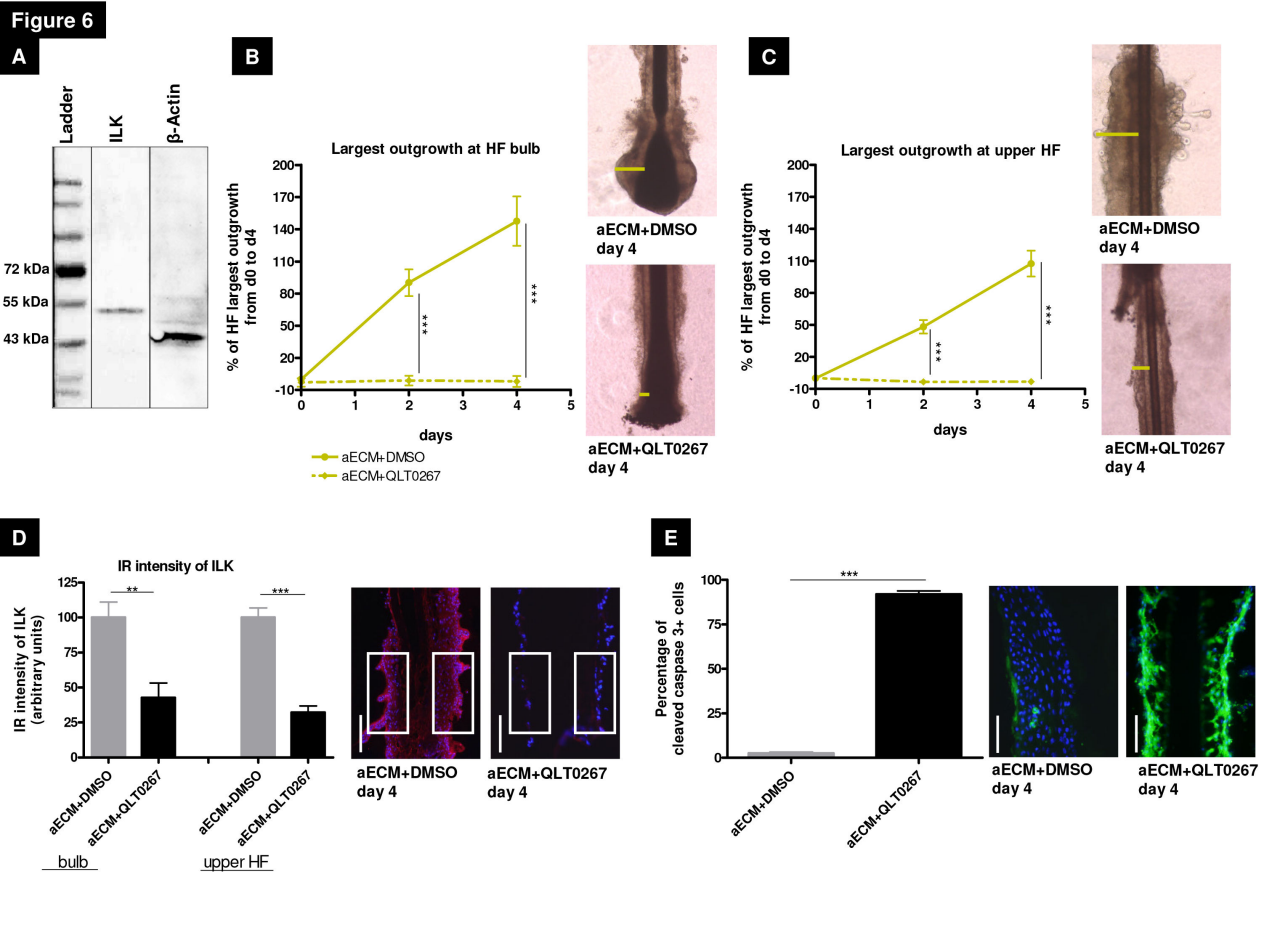
Integrin-linked kinase inhibition via QLT0267 stops the epithelial outgrowth. (A) Integrin-linked kinase (ILK) is expressed in human dispase-pretreated HF (hair follicle) keratinocytes, which was demonstrated by using the Western blot method in comparison to the protein expression of β-actin. (B) The analysis of the largest outgrowth in the HF bulb and (C) in the upper HF revealed the strong inhibitory effect of the pharmacological substance QLT0267 for the proliferative and migrative capacity. Representative photos. n=24-28 HFs of 3 individuals. (D) In the HF bulb and in the upper HF a significant reduction of ILK immunoreactivity could be demonstrated with QLT0267 treatment in comparison to our control HFs, which were dispase-pretreated and embedded in the artificial extracellular matrix with DMSO. Representative photos show the reference areas in the upper HF, n=7-10 HFs of 3 individuals. (E) The QLT0267 treatment caused a tremendous apoptotic effect in the HF epithelium, showing by nearly 100% cleaved caspase 3+ outer root sheath keratinocytes. n=5-10 HFs of 3 individuals. For each immunoreactivity intensity analysis the control was normalized to 100%. White scale bars=50µm. All statistical analyses were done by using Mann-Whitney test, (*p<0.05, **p < 0.01, ***p < 0.001); mean+/-SEM. Abbreviation: aECM+DMSO=artificial ECM consisting of Matrigel®, collagen I and K-SFM (keratinocyte-serum free medium), aECM+QLT0267=aECM supplemented with the 100µM pharmacological inhibitor QLT0267, DMSO=Dimethyl sulfoxide, ILK=integrin-linked kinase, IR=immunoreactivity.

As a first step towards dissecting the mechanisms by which ß1 integrin-mediated signaling impacts on human ePCs and their progeny *in situ*, we used the putative ILK inhibitor QLT0267 [38,43] to probe the role of ILK. This cytoplasmic adaptor protein of ß1 and ß3 integrin plays a key role in many ß1 integrin-mediated cellular processes, including actin rearrangement, cell adhesion, migration, proliferation, apoptosis and differentiation by associating with different regulatory proteins [40,75–78]. Since ILK protein expression has not yet been demonstrated in human HFs, this was first tested by Western blot. Indeed, human dispase-pretreated HF epithelium expressed ILK protein as the expected 53kDa band (Figure **6A**).

Using QLT0267, we examined whether blocking ILK affected human HFs *in situ*, focussing on ORSK survival and migration. HF epithelium was embedded in aECM, supplemented with 100µM QLT0267. Already during HF culture it became evident that approximately 40% of the aECM-embedded and QLT0267-treated HFs lost their adhesion to the Matrigel^®^/collagen milieu after 4 days of culture, a first overt evidence for massively reduced ILK activity to create FAs. The reduction of ILK expression via QLT0267 (100µM) was further documented by analyzing ILK protein IR at day 4. Both in the HF bulb and in the upper HF QLT0267 induced a significant reduction of ILK IR (by 60-70%) (Figure **6D**). Already 2 hours after QLT0267 incubation, the isolated HFs demonstrated a slightly reduction in ILK IR (Figure **S2D**). 

Furthermore, QLT0267 treatment also induced substantial HF dystrophy, and almost abolished both ORSK outgrowth (Figure **6B,C**) as well as cortactin-activated migration (Figure **S3**). This demonstrated that human ORSK migration *in situ* and F-actin cytoskeleton remodelling [77] critically depend on ILK-mediated signaling via a Src activation of proteins like cortactin, which is mandatory for their phosphorylation and thereby for actin assembly [79].

Moreover, DAPI staining revealed numerous pyknotic nuclei in the HF epithelium indicating the high level of HF dystrophy and apoptosis induced by QLT0267 treatment (data not shown). This was confirmed by quantitative immunohistomorphometry for cleaved caspase 3 (Figure **6E**), TUNEL and Ki-67, which documented massive intraepithelial apoptosis and cessation of ORSK proliferation (Figure **S2E**). Therefore, pharmacological inhibition of ILK likely induced anoikis, i.e. cell death due to a loss of connection with the ECM or adjacent cells [80,81], thereby destroying the entire ORS.

In summary, our studies provide the first evidence in a human complex system that ILK-dependent β1 integrin-mediated signaling is mandatory for the adhesion of basal layer ORSK to the ECM thus stabilizing cell-ECM connection via FAs as well as promoting survival of human HF epithelium. 

## Discussion

Most data on the function of β1 integrin-mediated signaling in ePCs and their interaction with the ECM are based on murine models [82–85] or cell culture experiments [66,86,87]. However, the role of β1 integrin-mediated signaling in human ePCs growing within their natural tissue habitat had been largely unknown. Using organ-cultured human scalp HFs as a clinically relevant model for studying human ePCs *in situ*, we show here that β1 integrin signaling controls survival, adhesion, and migration in distinct ORS populations, including human HF eSCs and their progeny. Moreover, our data suggest that ß1 integrin signaling is fundamental for maintenance of the HF bulge eSC niche, while different human HF ePC subpopulations differ in their response to ß1 integrin signaling. The examined HF effects of ß1 integrin signaling are ILK-dependent.

Though our method of siRNA *β1 integrin* silencing was functionally effective and was documented on the mRNA level, we could not detect any change in the protein level 4 days after transfection. While the half-life of integrins on the surface of cultured human keratinocytes *in vitro* reportedly is about 12 h [88], their half-life *in situ* and *in vivo* is much longer. For example, in murine epidermis, β1 integrin can still be detected *in vivo* 10 days after Cre activation and in some HFs β1 integrin IR is even visible after 1-2 weeks [3,89]. This likely explains the discrepancy of our mRNA and protein results after knock down. 

That β1 integrin might be needed as a niche receptor for regulating proliferation activity in distinct ePC populations in the HF bulb and bulge is in line with neonatal K5Cre β1 null-mice showing HF and sebaceous gland loss, and greatly reduced proliferation [89]. The current silencing results also are in line with our previous finding that the stimulation of ß1 integrin-mediated signaling enhances the proliferation of hair matrix keratinocytes in organ-cultured human HFs, using the ß1 integrin-activating antibody 12G10 [29].

K15 and CD200 are well accepted as ePC markers of the HF bulge [16,20,23,54,90], but whether the expression of these markers is ß1 integrin-dependent remains unclear. The current knock down data now clarify that ß1 integrin signaling is necessary for keeping human ePCs in an undifferentiated state *in situ*, i.e. for maintenance of K15^+^ and CD200^+^ ORSKs in the HF bulge. 

The role of β1 integrin in the maintenance of eSCs or ePCs is still controversially discussed. Jones and Watt proposed a role of β1 integrin signaling for the maintenance of human skin eSCs *in vitro*, because these cells expressed high levels of β1 integrin and showed typical SC properties like high CFE [28]. In contrast, a direct link between the loss of β1 integrin skin-specific conditional knock out to an ePC or eSC reduction could not be elucidated in mutant mice [84], and ß1 integrin protein levels *in situ* are not markedly higher in the human bulge than elsewhere in the human ORS [29]. However, here we show that β1 integrin silencing impacts on K15 and CD200 expression in a complex human mini-organ, the HF suggesting that β1 integrin-mediated signaling is indeed required for ePC maintenance in adult human HFs.

Since the human bulge likely represents an immunologically privileged SC niche [91], it is interesting that ß1 integrin silencing also reduces HF bulge expression of the immunoinhibitory “no danger”-signal, CD200. Future functional experiments, therefore, will need to clarify whether this reduced CD200 expression compromises the relative HF bulge immune privilege in human HFs. Clinically, this may be relevant for irreversible forms of human hair loss characterized by a loss of K15^+^/CD200^+^ bulge cells and a collapse of the HF bulge immune privilege [91], like the cicatricial alopecia, lichen planopilaris [61], where insufficient ß1 integrin-mediated signaling may contribute to the CD200-dependent component of the HF bulge immune privilege collapse demonstrated in this scarring hair loss disorder [91].

For the direct manipulation of β1 integrin-mediated signaling in ePC populations of the HF epithelium removal of the HFs BM and CTS by dispase appeared necessary. This, however, artificially disrupts cell-ECM connections and greatly dysregulates the surrounding ECM environment, likely inducing a broad range of abnormalities [92]. Under our HF organ culture conditions, this unphysiological culture of denuded HF epithelium is further compounded by the absence of serum components such that may promote ß1 integrin signaling, thus severely compromising the normal conditions for outgrowth and survival of the HF epithelium. To optimize this defective ECM environment, we used only Matrigel^®^ for embedding the HF epithelium, guided by previous work [93]. However, after several days, most HFs lost their adhesion to the provided surrogate matrix. This might result from the activity of enzymes like matrix metalloproteinase (MMPs) which are expressed in HFs for degrading ECM components and by this contribute to HF growth and cycling [94,95]. But such enzymes require a physiological balance between their activity and their specific inhibitors for a controlled function [94]. Adding collagen I to Matrigel^®^ (1:1) apparently mimicked the lost ECM signals arisen from the HFs CTS and BM, and enabled human HF ORSK outgrowth *in situ* and ORSK emigration. 

The opposite effects on proliferation and apoptosis in the HF bulb and bulge, but also the reduction of the ePC markers K15 and CD200 in these embedded HFs, confirm the widely appreciated effect of Matrigel^®^ as a stimulator of proliferation and differentiation [96]. Thus, this mouse sarcoma derived matrix is not an optimal surrogate of the HF ECM for mimicking the human SC niche *in situ* and for keeping human ePCs in an undifferentiated state. Therefore, better-defined human-derived alternative ECM composites are urgently needed [97,98].

Interestingly, inhibiting or activating β1 integrin signaling modulates the functions of human ePCs and their progeny in a highly differential manner, depending on where these cells are located within the HF epithelium. This obvious difference could not be shown measuring the whole outgrowth area of the HF (Figure **3A**) while the more specific analysis methods of the largest outgrowth and the proliferation/apoptosis of defined HF compartments (Figure **3C,D** and Figure **5C,D**), revealed the different antibody effects. These specific differences are possibly due to the functionally different cells types located in these compartments, namely ePCs in the HF bulge versus transit amplifying cells in the HF bulb. These findings also fit well to the analysis of the different proliferation and apoptosis potential in the bulb and the upper HF bulge.

The activating β1 integrin antibody (12G10) induces proliferation and differentiation in the HF bulb whereas the inhibiting β1 integrin antibody (mAb13) keeps the ePCs in a more undifferentiated state. But the opposite effects were shown on the more distal located HF compartment the upper HF including the bulge (see Figure **4** **for details**). Our results obtained with manipulating β1 integrin activity via specific activating and inhibiting antibodies arose new functions for their usage and invite the hypothesis that β1 integrin receptor-mediated signaling in HF matrix cells primarily regulates and stimulates the proliferative capacity and differentiation of the HF. In contrast, β1 integrin signaling in the eSC niche (HF bulge) appears to operate as quiescence signal by outside-in signaling via the surrounding ECM. By using the inhibiting antibody mAb13 this β1 integrin-mediated signaling in the hair matrix cells was changed followed by a tremendous apoptosis, whereas the proliferation quiescence function in the HF bulge altered which lead to an increased Ki-67^+^ cell number. 

We postulate that the different antibodies are useful markers to distinguish between ePCs in the bulge and transit amplifying cells in the bulb, which are known to have different proliferation capacities. While the transit amplifying cells of the HF bulb are highly active cells for reproducing/maintenance the HF, the ePCs of the HF bulge represent a slow cycling and quiescent ORSK population. Changes of the β1 integrin-mediated signaling in these HF compartments, for example by using of mAb13, lead to a disturbed outside-in signaling reaction. Consequently, mAb13 pushed the proliferating active transit amplifying cells into apoptosis, while the slow-cycling ePCs in the HF bulge did not change their status of activity. This novel finding shows that using these specific β1 integrin antibodies initiates different reactions of ePCs in the bulge and in the bulb. The manipulation of the β1 integrin-mediated signaling certainly appears to be one of the means by which the surrounding ECM profoundly and differentially modulates epithelial cell behavior in human HFs. 

The manipulation of the β1 integrin-mediated signaling certainly appears to be one of the means by which the surrounding ECM profoundly and differentially modulates epithelial cell behavior in human HFs. 

It is controversially debated whether ILK really is a true kinase or just a scaffolding protein [76,99–102] and it has been questioned how the pharmacological inhibitor QLT0267 really works, which was developed to inhibit ATP binding of ILK [44]. Until now the role of ILK in cellular processes has been studied in transformed and/or tumorigenic cells [37,38,103], and in mouse models [39,78,104], but not in a complex human mini-organ. By using our experimental setup an efficient reduction of ILK expression *in situ* via QLT0276, the potent apoptosis-inducing capacity [37,38] and the loss of adhesion (abrogates ORSK migration) was striking in pharmacologically ILK-blockaded human HFs (Figure **6**, Figure **S2E**). Former studies with this pharmacological inhibitor also demonstrated an effective reduction of ILK activity as well as a decrease of AKT (AKT kinase) and FAK (focal adhesion kinase) phosphorylation in tumor cell lines [36,38,44]. 

Importantly, our human HF organ culture data are in line with the results obtained in ILK-K5 knock out mice concerning the impaired directional migration followed by a missing forming of stable lamellipodia-like structures, as well as the detachment through the surrounding environment [105]. Thus, the high level of ORSK apoptosis could be caused by missing AKT phosphorylation after QLT0267 treatment, as previously described in a tumor cell line [41] or the impaired formation of FA because of a reduced ILK expression [106,107]. Irrespective of this consideration, our data suggest that ILK protein is functionally important for β1 integrin-mediated signaling in the human HF and for the survival of human ORSKs.

Taken together, our study demonstrates that ePCs in human HFs require β1 integrin-mediated signaling for survival, adhesion, and migration, and that different human HF ePC subpopulations differ in their response to β1 integrin signaling. Mechanistically, this effect is likely ILK-dependent. These new insights into the β1 integrin-dependence of distinct human ePC populations enrich our as yet very fragmentary understanding of the integrin-dependent topobiology of human ePCs *in situ*, and are relevant for cell-based regenerative medicine strategies that employ HF-derived ePCs [90,108].

## Supporting Information

Figure S1 **Differentiation of epithelial progenitor cells is regulated by β1 integrin ligands.** (A) Embedding into the niche mimicking aECM (artificial extracellular matrix) system significantly downregulated the gene expression of the HF (hair follicle) progenitor marker Keratin 15 (K15). The β1 integrin inhibiting antibody mAb13 increased the *K15* transcription.
(B) Embedding into the aECM system β1 integrin antibodies significantly reduced the gene expression of CD200. (C) The Keratin 6 (K6) transcription was strongly repressed in the aECM and aECM+12G10-treated group, while this reduction is not so high in the mAb13-treated group. n=1 (2) individuals in experimental triplicates (12-15 HFs). (D) Immunoreactivity of K15 was only found in the upper HF including the bulge. By counting K15+ cells in a specified area (250x125) the decrease of this progenitor marker was measurable. n=7-15 HFs of 3-4 individuals. (E) CD200+ cells were also only found in the upper HF including the bulge. The CD200+ cells were counted in a specified area (250x125) and confirmed the gene expression results of this progenitor marker. n=7-10 HFs of 2-3 individuals. (F) The immunoreactivity expression pattern of K6 was analysed in the HF bulb, lower HF and upper HF by quantitative immunohistochemistry in fixed rectangle. The supplementation of the inhibitory antibody mAb13 reduced the differentiation inducing capacity of the artificial ECM system in the whole HF. n=4-7 HFs of 3 individuals. White scale bars in the representative photos=100µm. All data were analysed by using the One way ANOVA, Bonferroni post hoc test, mean +/- SEM (*p<0.05, **p<0.01, ***p<0.001). Abbreviation: aECM = artificial ECM consisting of Matrigel^®^, collagen I and K-SFM (keratinocyte-serum free medium), aECM+12G10=aECM supplemented with the activating β1 integrin antibody 12G10, aECM+mAb13=aECM supplemented with the inhibiting β1 integrin antibody mAb13, HKG=housekeeping gene, PPIA=peptidylprolyl isomerase A.
(TIF)

Figure S2 **β1 intergin knock down and the inhibition via QLT0267.** (A) The knock down of β1 integrin did not alter the CD71 immunoreactivity (IR) expression in the HFs. n=11-18-HFs of 2 individuals. (B) MHC Ia IR intensity demonstrated that the silencing reaction and the specific knock down of ITGβ1 had no influence on the immune privilege of the HF bulge. n=12-19 HFs of 2 individuals. (C) Representative photos of the EdU IR of scrambled control and ITGβ1 KD HFs are shown. (D) Intergin-linked kinase (ILK) IR intensity in human HFs after 2h incubation in 37°C with or without QLT0267 before embedding into the artificial extracellular matrix demonstrated the fast reduction/inhibiton of ILK in outer root sheath keratinocytes. (E) Ki-67/TUNEL-staining confirmed the caspase 3 staining, while the QLT0267-treated HFs showed no proliferation, but a high apoptosis rate in comparison to the DMSO-treated control. Red bars=Ki-67, green bars=TUNEL. n=7-12 HFs of 3 individuals. For each analysis of the IR intensity the control was normalized to 100%. White scale bars=50µm. All statistical analyses were done by using Mann-Whitney test, (***p<0.001), mean +/- SEM. Abbreviation: ITGβ1 KD = *β1* *integrin* knock down, MHC Ia = major histocompatibility complex (MHC) class Ia.
(TIF)

Figure S3 **Integrin-linked kinase inhibition via QLT0267 inhibits keratinocyte migration.** The cortactin immunoreactivity was nearly absent in the aECM+QLT0267-treated HFs compared to the aECM+DMSO-treated group on day 4. This demonstrated that human outer root sheath keratinocyte migration *in* *situ* and F-actin cytoskeleton remodelling [76] depend on integrin-linked kinase (ILK)-mediated signalling via a Src (Proto-oncogene tyrosine-protein kinase) activation of proteins like cortactin. Abbreviation: aECM+DMSO=artificial ECM consisting of Matrigel^®^, collagen I and K-SFM (keratinocyte-serum free medium), aECM+QLT0267=aECM supplemented with the 100µM pharmacological inhibitor QLT0267, DMSO=Dimethyl sulfoxide.
(TIF)

Text S1 (DOC)

Text S2 (DOC)
