# Correction: β1 Integrin Signaling Maintains Human Epithelial Progenitor Cell Survival In Situ and Controls Proliferation, Apoptosis and Migration of Their Progeny

**DOI:** 10.1371/annotation/bd34416c-34be-4864-aa51-cd855ee35b9c

**Published:** 2014-01-17

**Authors:** Nancy Ernst, Arzu Yay, Tamás Bíró, Stephan Tiede, Martin Humphries, Ralf Paus, Jennifer E. Kloepper

This article was republished on January 13th, 2014 to correct for errors in multiple figures and legends in the original version of the article (which can be viewed here: http://www.plosone.org/corrections/pone.0084356.001.cn.pdf).

