# Supplementary figures and images for: β1 Integrin Signaling Maintains Human Epithelial Progenitor Cell Survival In Situ and Controls Proliferation, Apoptosis and Migration of Their Progeny
Source: PLoS One. 2013 Dec 27;8(12):e84356. doi: 10.1371/journal.pone.0084356 (PMC3874009; doi:10.1371/journal.pone.0084356)

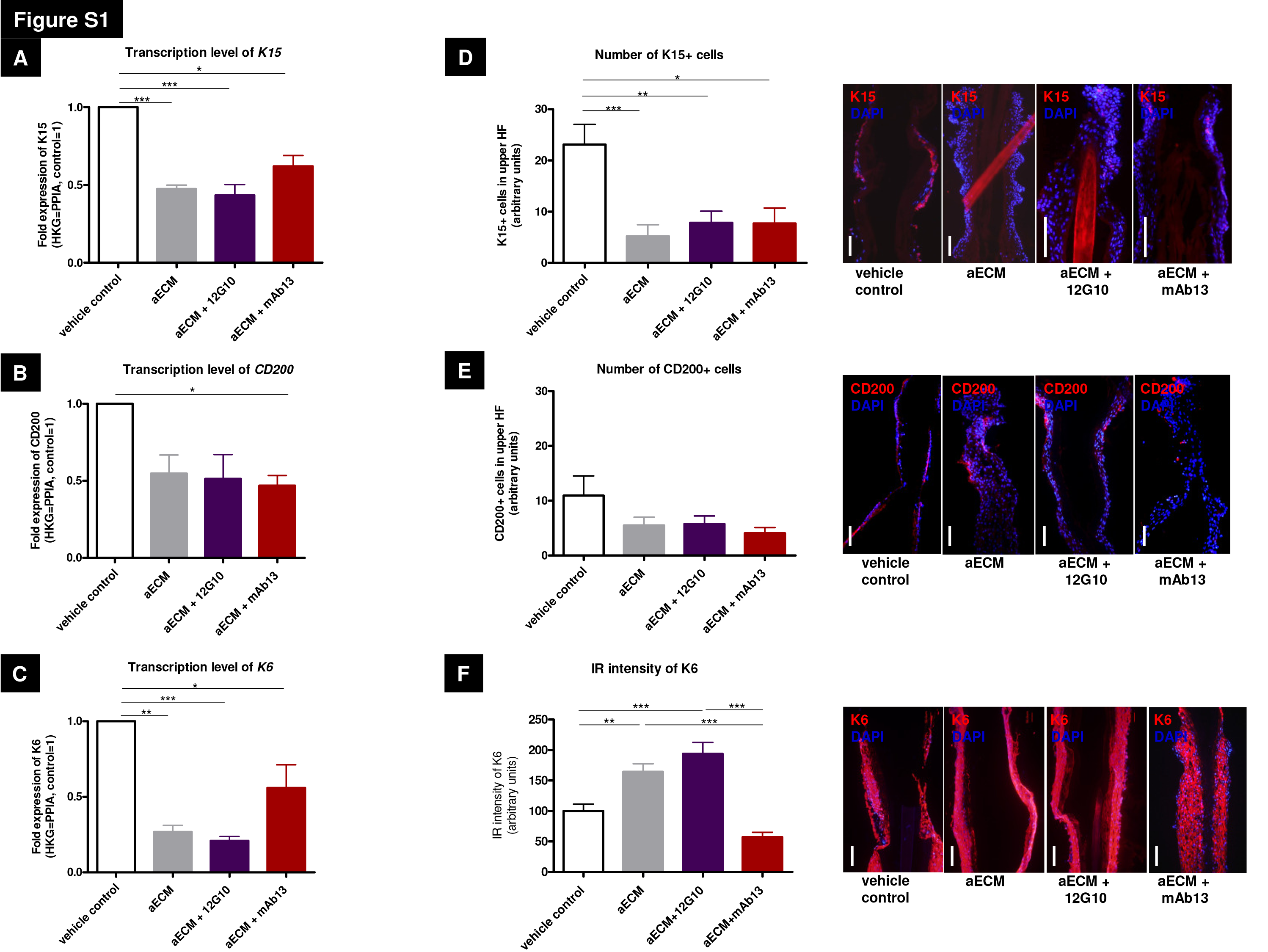

Supplement: Figure S1 — Differentiation of epithelial progenitor cells is regulated by β1 integrin ligands. (A) Embedding into the niche mimicking aECM (artificial extracellular matrix) system significantly downregulated the gene expression of the HF (hair follicle) progenitor marker Keratin 15 (K15). The β1 integrin inhibiting antibody mAb13 increased the K15 transcription. (B) Embedding into the aECM system β1 integrin antibodies significantly reduced the gene expression of CD200. (C) The Keratin 6 (K6) transcription was strongly repressed in the aECM and aECM+12G10-treated group, while this reduction is not so high in the mAb13-treated group. n=1 (2) individuals in experimental triplicates (12-15 HFs). (D) Immunoreactivity of K15 was only found in the upper HF including the bulge. By counting K15+ cells in a specified area (250x125) the decrease of this progenitor marker was measurable. n=7-15 HFs of 3-4 individuals. (E) CD200+ cells were also only found in the upper HF including the bulge. The CD200+ cells were counted in a specified area (250x125) and confirmed the gene expression results of this progenitor marker. n=7-10 HFs of 2-3 individuals. (F) The immunoreactivity expression pattern of K6 was analysed in the HF bulb, lower HF and upper HF by quantitative immunohistochemistry in fixed rectangle. The supplementation of the inhibitory antibody mAb13 reduced the differentiation inducing capacity of the artificial ECM system in the whole HF. n=4-7 HFs of 3 individuals. White scale bars in the representative photos=100µm. All data were analysed by using the One way ANOVA, Bonferroni post hoc test, mean +/- SEM (*p<0.05, **p<0.01, ***p<0.001). Abbreviation: aECM = artificial ECM consisting of Matrigel®, collagen I and K-SFM (keratinocyte-serum free medium), aECM+12G10=aECM supplemented with the activating β1 integrin antibody 12G10, aECM+mAb13=aECM supplemented with the inhibiting β1 integrin antibody mAb13, HKG=housekeeping gene, PPIA=peptidylprolyl isomerase A. (TIF) [file pone.0084356.s001.tif]

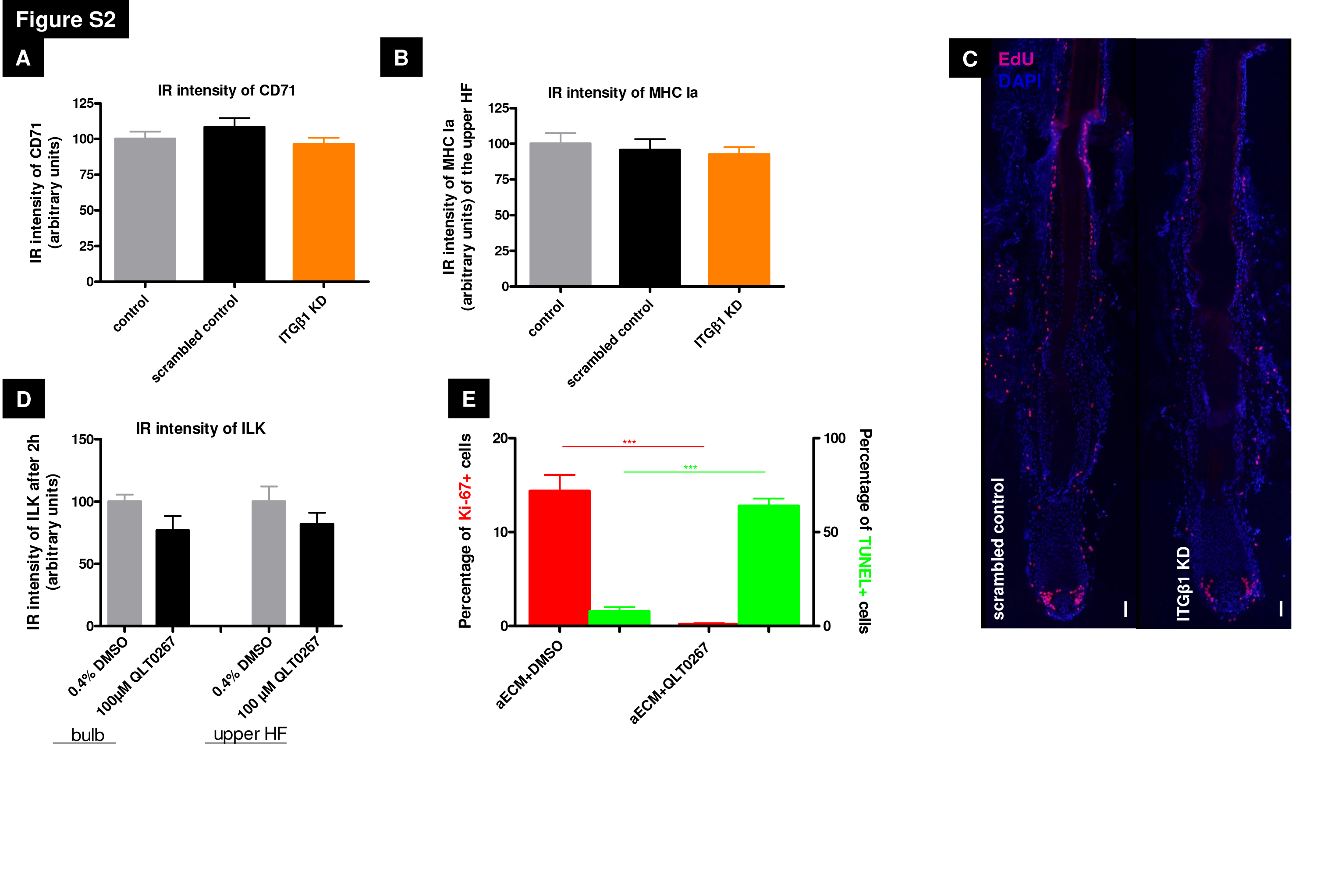

Supplement: Figure S2 — β1 intergin knock down and the inhibition via QLT0267. (A) The knock down of β1 integrin did not alter the CD71 immunoreactivity (IR) expression in the HFs. n=11-18-HFs of 2 individuals. (B) MHC Ia IR intensity demonstrated that the silencing reaction and the specific knock down of ITGβ1 had no influence on the immune privilege of the HF bulge. n=12-19 HFs of 2 individuals. (C) Representative photos of the EdU IR of scrambled control and ITGβ1 KD HFs are shown. (D) Intergin-linked kinase (ILK) IR intensity in human HFs after 2h incubation in 37°C with or without QLT0267 before embedding into the artificial extracellular matrix demonstrated the fast reduction/inhibiton of ILK in outer root sheath keratinocytes. (E) Ki-67/TUNEL-staining confirmed the caspase 3 staining, while the QLT0267-treated HFs showed no proliferation, but a high apoptosis rate in comparison to the DMSO-treated control. Red bars=Ki-67, green bars=TUNEL. n=7-12 HFs of 3 individuals. For each analysis of the IR intensity the control was normalized to 100%. White scale bars=50µm. All statistical analyses were done by using Mann-Whitney test, (***p<0.001), mean +/- SEM. Abbreviation: ITGβ1 KD = β1 integrin knock down, MHC Ia = major histocompatibility complex (MHC) class Ia. (TIF) [file pone.0084356.s002.tif]

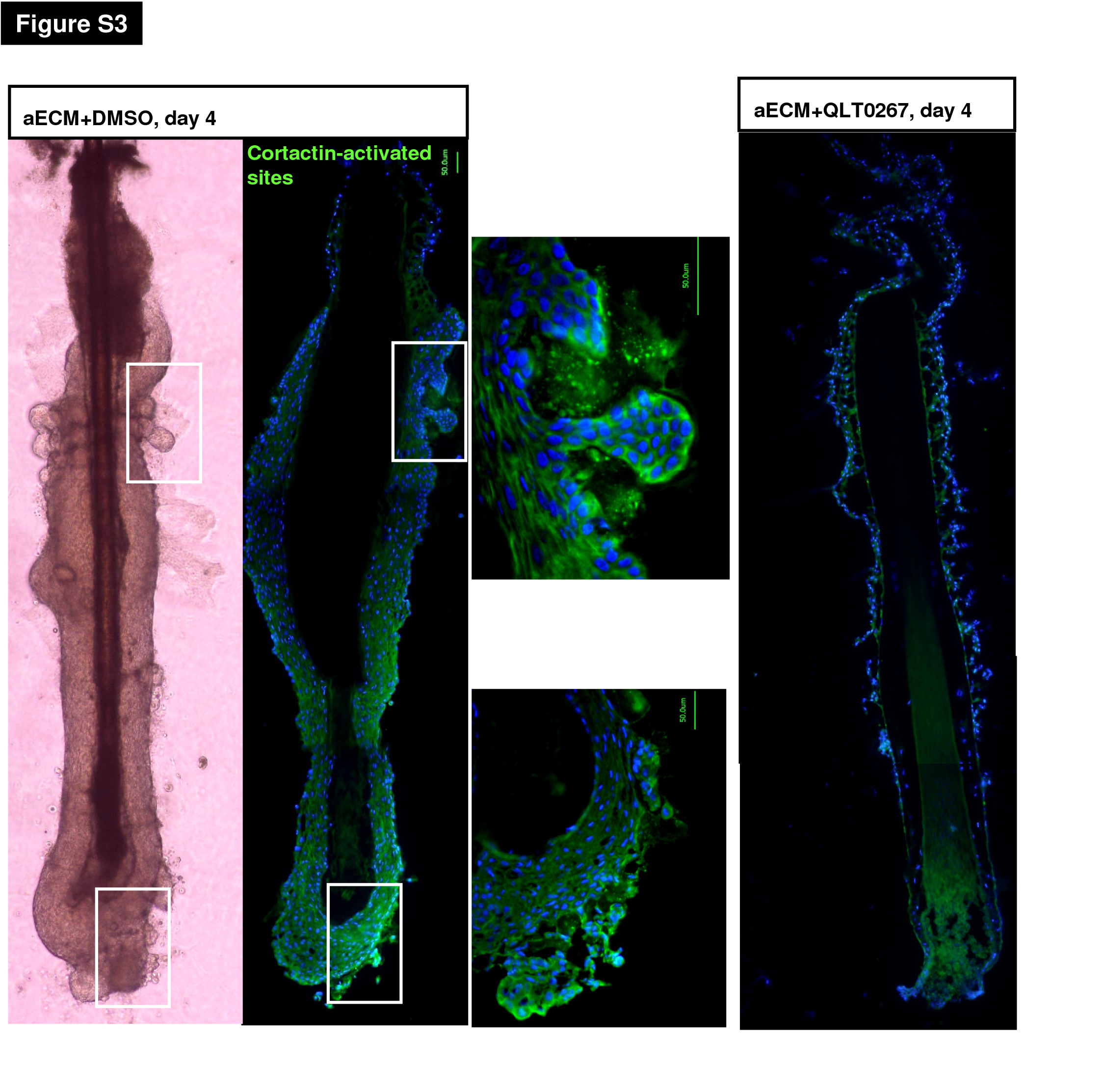

Supplement: Figure S3 — Integrin-linked kinase inhibition via QLT0267 inhibits keratinocyte migration. The cortactin immunoreactivity was nearly absent in the aECM+QLT0267-treated HFs compared to the aECM+DMSO-treated group on day 4. This demonstrated that human outer root sheath keratinocyte migration in situ and F-actin cytoskeleton remodelling [76] depend on integrin-linked kinase (ILK)-mediated signalling via a Src (Proto-oncogene tyrosine-protein kinase) activation of proteins like cortactin. Abbreviation: aECM+DMSO=artificial ECM consisting of Matrigel®, collagen I and K-SFM (keratinocyte-serum free medium), aECM+QLT0267=aECM supplemented with the 100µM pharmacological inhibitor QLT0267, DMSO=Dimethyl sulfoxide. (TIF) [file pone.0084356.s003.tif]
