# Supplementary material for: β1 Integrin Signaling Maintains Human Epithelial Progenitor Cell Survival In Situ and Controls Proliferation, Apoptosis and Migration of Their Progeny
Source: PLoS One. 2013 Dec 27;8(12):e84356. doi: 10.1371/journal.pone.0084356 (PMC3874009; doi:10.1371/journal.pone.0084356)
Supplement: Text S1 — (DOC) [file pone.0084356.s004.doc]

**SUPPLEMENTARY TEXT 1**

Integrin conformation change corresponding activity has been analyzed with monoclonal antibodies (mAbs) that detect conformation-dependent epitopes [109] and can affect the functions of this receptor. Some mAbs against binding β1 integrins that recognize ligand-induced receptor binding sites, stimulate the receptor activity, possibly by stabilizing the ligand-occupied conformation of the integrin [65] and by inducing the clustering of cell-surface integrins, and the preferential localization of β1 integrins expressing the 12G10 epitope at cell-cell adhesion sites [110], like the activating antibody 12G10.

These activating 12G10 and inhibiting mAb13 antibodies are ligand-mimicking RGD peptides and showed an influence on hair follicle (HF) elongation and proliferation [29]. Moreover, different studies proved the specific function of these antibodies in cell culture, for example mAb13 acts as an allosteric inhibitor and reduces ligand-binding leading to a displacement of the ligand or the loss of cell adherence [65,66,111]. Furthermore the anti-β1 integrin mAb13 inhibits the glandular differentiation of SW1222 cells (61%) and their cellular binding to type I collagen (60%) [112].

**SUPPLEMENTARY REFERENCES**

**109.** **Byron, A., Humphries, J.D., Askari, J.A., Craig, S.E., Mould, A.P., Humphries, M.J.** (2009). Anti-integrin monoclonal antibody. *J Cell Sci*

**110. Whittard J.D., Akiyama S.K.** (2001). Positive regulation of cell-cell and cell-substrate adhesion by protein kinase A. *J Cell Sci*

**111. Strobel T., Cannistra S.A.** (1999). Beta1-integrins partly mediate binding of ovarian cancer cells to peritoneal mesothelium in vitro. *Gynecol Oncol.*

**112. Pignatelli M., Liu D., Nasim M.M., Stamp G.W., Hirano S., Takeichi M.** (1992). Morphoregulatory activities of E-cadherin and beta-1 integrins in colorectal tumour cells. *Br J Cancer*
