# Supplementary material for: β1 Integrin Signaling Maintains Human Epithelial Progenitor Cell Survival In Situ and Controls Proliferation, Apoptosis and Migration of Their Progeny
Source: PLoS One. 2013 Dec 27;8(12):e84356. doi: 10.1371/journal.pone.0084356 (PMC3874009; doi:10.1371/journal.pone.0084356)
Supplement: Text S2 — (DOC) [file pone.0084356.s005.doc]

**SUPPLEMENTARY TEXT 2**

We have already shown a strong effect on the differentiation-inducing capacity of our extracellular matrix (ECM) environment alone. Next, we want to clarify the influence of additional manipulation of β1 integrin-mediated signaling via specific antibodies. Supplementation of β1 integrin inhibiting antibody mAb13 to HFs embedded in the ECM system slightly increased again the *Keratin15* *(K15)* gene expression (**Fig.S1A**), whereas 12G10 demonstrated no strong influence on the epithelial progenitor cell markers K15 and CD200 gene and protein expression (**Fig.S1A-B,D-E**). The analysis of the K6 gene and protein expression displayed a biphasic signaling effect like in the vehicle control. But it seemed that these two β1 integrin antibodies acted as opponents in the K6 expression independently from the investigation of the gene or protein expression (**Fig.S1C,F**). Interestingly, the β1 integrin inhibiting antibody mAb13 enhanced the *K6* gene expression (**Fig.S1C**), but lead to a K6 immunoreactivity decrease in the whole HF (**Fig.2F**), whereas the 12G10 treatment displayed the same expression pattern like the vehicle control.
